# Toxicity and Metal Corrosion of Glutaraldehyde-Didecyldimethylammonium Bromide as a Disinfectant Agent

**DOI:** 10.1155/2018/9814209

**Published:** 2018-07-09

**Authors:** Wenshu Lin, Bing Niu, Jialin Yi, Zhirui Deng, Jiang Song, Qin Chen

**Affiliations:** Shanghai Key Laboratory of Bio-Energy Crops, School of Life Sciences, Shanghai University, Shanghai, China

## Abstract

The wide use of disinfectants has prompted resistance from the microbiome which will in turn reduce the bactericidal effect of disinfectants. Hence, glutaraldehyde (GA) and didecyldimethylammonium bromide (DDAB) were used to develop a combination disinfectant with high stability and antimicrobial effects, which was named GA-DDAB combination disinfectant (GD). The bactericidal mechanism against* Escherichia coli* was studied in our earlier work. In this study, we focused on GD's bactericidal efficacy in both the laboratory and environment, the genetic toxicity to mouse lymphoma L5178Y* TK*^+/−^ cells, acute peroral toxicity in mice, and its metal corrosion properties with a view to providing theoretical support for developing a high-efficiency, low toxicity, and weakly corrosive disinfectant for general use.

## 1. Introduction

Increasing trade among countries worldwide has promoted and expanded the development of the international maritime transportation industry. Networks of container transportation for trade have advanced between global ports. The surfaces of containers are polluted with microorganisms during transportation from different ports [1, 2]. Pathogenic microorganisms such as viruses, bacteria, and fungi threaten human health and agriculture and can lead to significant economic losses [3, 4].

Disinfectants play an important role in preventing infection by pathogens. However, environmental and biotic factors including temperature, organic interfering substances, and a diverse microbial species affect the bactericidal activities of disinfectants [5, 6]. Disinfectants might get rapidly diluted after application in the environment [7, 8], and pathogens may attain resistance and cross-resistance through phenotypic adaptation or genetic inheritance [9, 10]. The acquisition of resistance is commonly attributed to incomplete disinfection, adding to the risk of contamination infection.

The aim of this study was to develop an efficient and safe combination disinfectant for the disinfection of shipping containers and prevent infectious outbreaks. Glutaraldehyde (GA) is widely used in the industrial, scientific, and biomedical fields [11] and didecyldimethylammonium bromide (DDAB) belongs to a class of quaternary ammonium compounds (QACs) and is used commonly as a disinfectant. A combination disinfectant of GA and DDAB was developed in our previous work. The formulation (GD) was found to have high stability and potent antimicrobial effects. Here, the bactericidal efficacy, safety, potential field applications, and metal corrosive properties of GD were further investigated.

## 2. Materials and Methods

### 2.1. Materials

The five strains of microorganisms used in this study were* Escherichia coli *ATCC 25922,* Staphylococcus aureus* ATCC 6538,* Pseudomonas aeruginosa* ATCC 15442,* Candida albicans* ATCC 10231, and* Aspergillus niger *ATCC 16404.

Mouse lymphoma L5178Y* TK*^*+/-*^ cells, clone 3.7.2C, were purchased from the Cell Bank Type Culture Collection of the Chinese Academy of Science. RPMI1640 medium, heat-inactivated horse serum, penicillin, and streptomycin were purchased from Thermo Fisher Scientific (Waltham, Massachusetts, USA). The mammalian liver postmitochondrial fraction, S9, was purchased from Moltox (Molecular Toxicology, Inc., Boone, NC, USA). All other chemicals and reagents were of the highest grade available and were purchased from Sigma-Aldrich (St. Louis, MO, USA).

Female mice (4 weeks of age, specific pathogen-free; experimental animal use license number: SCXK [Hu] 2016-0003) were purchased from Shanghai Yukun Laboratory Animals Co. Ltd. The experiments were approved by the Animal Experiments Committee of the School of Life Science, Shanghai University, China.

Two disinfectants used in this study were 25% GA solution (CAS111-30-8; order no. A500484; Sangon Biotech Co. Ltd., Shanghai, China) and 80% DDAB solution (lot no. A1325004; Aladdin Industrial Corporation, Shanghai, China).

U, a combination of 0.022% didecyldimethylammonium chloride and 0.02% methenamine, was chosen as a positive control to evaluate the bactericidal effect of GD.

### 2.2. Bactericidal Efficacy Assays

The numbers of bacterial suspensions of* E. coli*,* S. aureus*,* P. aeruginosa*,* C. albicans*, and* A. niger* were 5 × 10^8^ CFU mL^−1^, 5 × 10^8^ CFU mL^−1^, 5 × 10^8^ CFU mL^−1^, 5 × 10^7^ CFU mL^−1^, and 5 × 10^7^ CFU mL^−1^, respectively. The experimental methods were suspension quantitative germicidal tests and were as described previously [12].

### 2.3. Comparison of Bactericidal Efficacy Assays

The numbers of bacterial suspensions of* E. coli *was 5 × 10^8^ CFU mL^−1^. Treatment time was 0, 10 and 20 minutes (min). The experiment methods were suspension quantitative germicidal tests and were also same as in our previous study [12]. The final concentration of GD was 0.005% GA and 0.001% DDAB. All the tubes and solutions were sterilized previously and the experiments were performed in a biological safety cabinet. Negative control was tryptone saline solution buffer.

### 2.4. Bactericidal Test of GD against Microbe in Environment

Four empty shipping containers (internal length: 11.89 m, internal width: 2.13 m, and internal height: 2.44 m) were randomly selected which had not been treated with disinfection or cleaning agents. Spray conditions were as follows: backpack sprayer, fog diameter size of 80–120*μ*m, spray distance of 5–10 cm, and spray amount of 100 mL m^−2^. On one side of the empty box, 6 adjacent spray areas of about 50 cm^2^ were selected as sampling points; each area was treated with water, GD (treat time: 2.5min), GD (treat time: 5min), GD (treat time: 10min), GD (treat time: 20min), and U (treat time: 20min), respectively. The samples were kept in neutralizer (5% lecithin, 5% Tween-80). At the scheduled time, 5 cm × 5 cm sterile plates and sterile cotton swabs were used for collecting samples [13]. The remaining 3 sides were treated by the same methods, and the procedure was also repeated for 3 other boxes, yielding a total of 16 samples for each disinfectant treatment. After sampling, the sample was diluted 10 times; 1 mL subsamples of the diluted solution were collected and cultured, and the microbes were counted. The killing rate was calculated from the following equation:

(1) $$\begin{array}{c} Killing\;\;rate=\frac{c_{1}-c_{2}}{c_{1}}\times 100\% \end{array}$$

where c_1_ is average number of colonies without disinfection treatment and c_2_ is average number of colonies after disinfection treatment.

### 2.5. DNA Extraction, PCR-Amplification of 16S rRNA and Internal Transcribed Spacer Gene Fragments, and DNA Sequencing

Two adjacent sampling points were selected on one side of the empty box and then sprayed with sterile water and GD (0.05% GA and 0.01% DDAB). After 2.5 min, the samples were collected with sterile plates and cotton swabs. The left 3 sides were subjected to the same procedures.

Microbial DNA was extracted from samples using E.Z.N.A. soil DNA kit (Omega Bio-tek, Norcross, GA, USA) according to the manufacturer's protocols.

The V3-V4 region of the bacterial 16S rRNA gene and fungal internal transcribed spacer were amplified by PCR. Bacterial primers: 338F (5'-ACTCCTACGGGAGGCAGCAG-3') and 806R (5'-GGACTACHVGGGTWTCTAAT-3') [14]. Fungal primers: ITS1F (5'-CTTGG TCATTTAGAGGAAGTAA-3') and ITS2-2043R (5'-GCTGCGTTCTTCATCGATGC-3') [15]. Purified amplicons were pooled in equimolar concentrations and paired-end sequenced (2× 300) on an Illumina MiSeq platform (Illumina, San Diego, USA) according to the standard protocols by Majorbio Bio-Pharm Technology Co. Ltd. (Shanghai, China).

### 2.6. Acute Peroral Toxicity Test

Different doses of GD were applied in this toxicity test (Table 2). Mice were treated with 0.2 mL for every 10 g weight by oral gavage. In addition, mice were fasted but allowed access to water for 4 h before the test, and a normal diet was provided after oral gavage. Weight and mortality were used to evaluate the toxicity of GD.

### 2.7. Genetic Toxicology Assays

The mouse lymphoma assay, which uses the kinase (*Tk*) gene as a target, is the most widely experimented mammalian cell gene-mutations. The details of this experiment were as previously described [16, 17]. Spontaneous mutation of* TK*^*-/-*^ genotype cells was confirmed before the test. The 4 groups were evaluated with final disinfectant concentrations of 50 *μ*g mL^−1^ GA + 10 *μ*g mL^−1^ DDAB, 100 *μ*g mL^−1^ GA + 20 *μ*g mL^−1^ DDAB, 150 *μ*g mL^−1^ GA + 30 *μ*g mL^−1^ DDAB, and 200 *μ*g mL^−1^ GA + 40 *μ*g mL^−1^ DDAB. The disinfectant treatment time was 3 hours (h). A mammalian liver postmitochondrial fraction (S9) with a final concentration of 2% was applied in the metabolic activation system, and the medium was used in the system without activation (no activation system). Plating efficiency (PE), relative survival (RS) rate, relative suspension growth (RSG), relative total growth (RTG), and mutation frequency (MF) were calculated.

Global Evaluation Factor (GEF) is defined as the mean plus one standard deviation based upon the distribution of the historical negative control data collected across laboratories. For the microwell version of the MLA the GEF is 126 × 10^−6^. For example, if the negative/solvent control MF in a microwell experiment is 50 ×10^−6^, one of the test cultures must have a MF of at least 50 + 126 = 176 × 10^−6^ to meet the GEF criterion for a positive call [18, 19].

### 2.8. Metal Corrosion Test

Generally, shipping containers are typically made of steel, aluminum, and stainless steel. The equipment in the casing of shipping containers may contain copper fittings. Thus, stainless steel, aluminum, and copper were selected for this study. Sheet metal (diameter: 24.0 mm, thickness: 1.0 mm, hole diameter: 2.0 mm) was degreased, dried (1 h at 50°C), cooled (room temperature), and weighed (precision of 0.1 mg). Each sample was immersed in 200 mL of water, 0.005% GA + 0.001% DDAB, and 0.05% GA + 0.01% DDAB for 72 h. The corrosive products were then removed and the sheets washed with water. A coarse filter paper for moisture removal was applied and the sheets were dried and weighed. The metal corrosion rate (R, mm·a^−1^) can be obtained as follows:

(2) $$\begin{array}{c} R=\frac{8.76\times 10^{7}\times \left(m-m_{t}\right)}{S\times t\times d} \end{array}$$

where m is the weight of the metal sheet before treatment (g), m_t_ is the weight of the metal piece after treatment (g), S is the total surface area of the metal sheet (cm^2^), t is the test time (h), and d is the density of the metal (kg m^−3^).

### 2.9. Statistical Analysis

The data are expressed using the SPSS 20.0 statistical program.* P*-values < 0.01 are considered highly significant at a 95% confidence level.

## 3. Results and Discussion

### 3.1. Bactericidal Effects of GD on Bacteria and Fungi

Bacteria and fungi exist everywhere in the environment. Pathogenic fungi can cause great harm to humans, plants, and animals, especially to critically ill patients [20].* Micrococcus*,* Staphylococcus*, and* Pseudomonas* have been shown to be the dominant bacteria in the air [21, 22]. These may threaten human health and agriculture and lead to significant economic losses. Therefore, 5 pathogenic microorganisms,* E. coli*,* P. aeruginosa*,* S. aureus*,* C. albicans*, and* A. niger*, were chosen in order to investigate the bactericidal and fungicidal effects of GD (Table 1). GD showed the strongest bactericidal activity against* E. coli.* However, slightly weaker antibacterial effects were also observed for* P. aeruginosa*,* S. aureus*, and* C. albicans*. The effective fungicidal concentrations for* A. niger* conidia were 0.075% GA and 0.015% DDAB after exposure for 40 min.* E. coli* was shown to be the more sensitive to GD than others, whereas* A. niger* conidia was much more tolerant.

The sensitivity of bacteria to GD is associated with the mode of action of the disinfectant and the cell structure of the organism. Studies have shown that GA undergoes strong crosslinking with proteins on the bacterial surface and inhibits the transport systems of gram-negative bacteria [23, 24]. DDAB is a cationic surfactant and belongs to a class of quaternary ammonium compounds (QACs), which functions to destroy the microbial cell membrane and change the phospholipid bilayer biochemically [25, 26]. Our previous study demonstrated that GD killed* E. coli* by damaging the cell wall and cytoplasmic membrane, causing intracellular component extravasation [12]. This is the main cause of GD sensitivity to* E. coli. *Meanwhile,* A. niger *conidia contain little water and have a complex surface structure, thereby impeding the entry of disinfectants into cells and hence warranting higher treatment times and higher concentrations in order to be effective.

**Figure 1:**
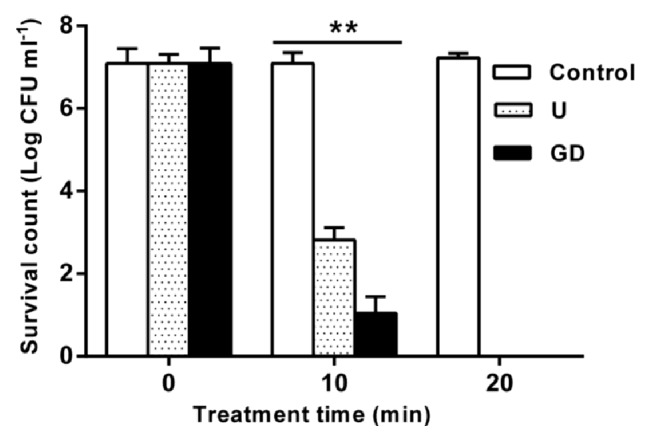
**Comparison of bactericidal efficacy of disinfectants on* E. coli***. Note: control without disinfectant treatment; U is a combination of 0.022% dimethyldimethylammonium chloride and 0.02% methenamine; GD is a combination of 0.005% GA and 0.001% DDAB. Treatment time is 0, 10, and 20 min. The means ± SD for at least three replicates are illustrated. *∗∗ P* < 0.01.

After determination of the effective bactericidal concentration, GD's bactericidal effects were evaluated by comparing its action with U against* E. coli *(Figure 1). After 10 min, the numbers of surviving* E. coli* cells were approximately 10 and 10^3^ CFU/mL for GD and U, respectively. The killing rates of GD and U were both 100% after exposure for 20 min. In addition, the quick-acting bactericidal effect of GD toward* E. coli* was higher than that of U. The results shows that GD was more effective than U as a bactericidal agent.

### 3.2. Bactericidal Effects of GD against Environmental Microbes

**Figure 2:**
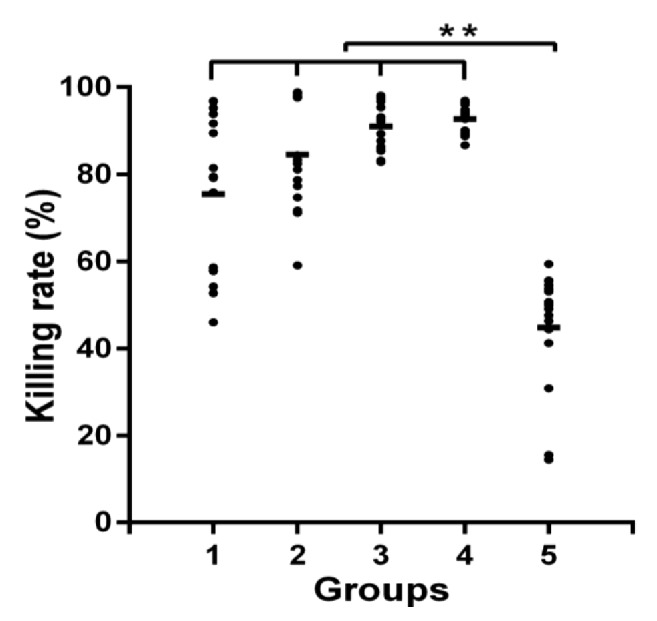
**Bactericidal effects of GD against environmental microbe**. Note: Groups 1-4 are a combination disinfectant of GD (0.05% GA + 0.01% DDAB) treated 2.5, 5, 10, and 20min, respectively; Group 5 was exposed in U after 20min; n = 16; *∗∗ P* < 0.01.

The bactericidal effects of GD at different times were investigated in shipping empty containers. As shown in Figure 2, the average killing rates were 75.5%, 84.6%, 91.0%, and 92.7% after exposure to GD for different times in an empty container. Bactericidal effects of GD against environmental microbes have great differences in initial short time (2.5min) but tend to be stable over longer time periods. However, the effective bactericidal concentration of GD in the environment was 5-fold that in the laboratory. This discrepancy might be explained as follows. First, microorganisms in the environment might show stronger resistance. Many microorganisms, such as* P. aeruginosa* and* S. aureus*, form biofilms, causing resistance to antibacterial agents [27, 28]. Second, the complexity of the environment decreases the bactericidal effects of disinfectants. After the application of disinfectants, the ambient temperature, humidity, wind, light, and other factors affect the bactericidal effects of the applied chemicals [5, 6, 29]. The average killing rate of U was 44.8% after 20 min, which was lower than that of GD. This indicated that GD had a high-efficiency and quick-acting bactericidal effect. The bactericidal efficacy of GD was better than that of U.

### 3.3. Effects of GD on Microbial Biomass and Community Structure

**Figure 3:**
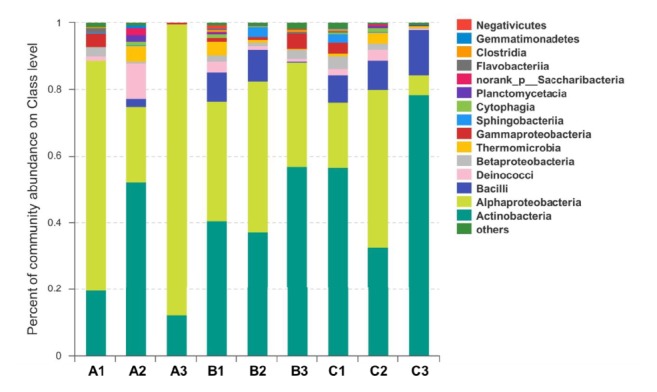
**Effect of GD to environmental microbial community diversity**. Note: A1, B1, and C1 are control without disinfectant treatment. A2, B2, and C2 are GD treatment for 2.5 min. A3, B3, and C3 are GD treatment for 10 min. This refers to three repetitions.

Analysis of microbial community diversity was assessed after the bactericidal effects of GD against environmental microbes. As shown in Figure 3, compared with GD and control, the bacterial community structure was seen to be different. The microbial community diversity changed little after treatment of GD for 2.5 min. The relative community abundance of* Actinobacteria*,* Alphaproteobacteria*, and* Bacilli* constitutes the main components after treatment with GD for 10 min, and the other 12 classes of bacteria were killed. This indicated that the above 3 classes of bacteria were found to be insensitive to GD. This suggests that GD can killed multiple types of bacteria effectively. In addition, high-throughput sequencing analysis verified that the effective bactericidal concentration of GD in the environment was 5-fold that in the laboratory. A variety of bacterial spores and moulds are highly resistant to disinfectants, and the multifactorial nature of the ambient environment might be the main cause of the increased disinfectant concentration required in the field compared with that needed in the laboratory for performing the same task.

### 3.4. Acute Peroral Toxicity of GD* In Vivo*

Mice were treated with GD and then observed for 14 days. Mortality rates are shown in Table 2. Notably, body weights decreased as the dose of GD used increased. In addition, the mortality rates were 70% in mice treated with 0.4% GA + 0.08% DDAB and 100% (within 24 h) in mice treated with 0.5% GA + 0.1% DDAB. Thus, high concentrations of GD showed acute peroral toxicity. Studies have shown that high concentrations of GA have different effects on the skin, eyes, and mucous membranes [30]. Ballantyne et al. also showed that GA elicits some acute toxicities [31]. Clinical observations have indicated that 0.5% GA is a slight irritant to humans [32]. A study by Marzulli and Maibach indicated that 34.4% of the studied subjects were sensitive to 5% GA [33]. In this study, our results showed that high concentrations of GD elicited symptoms of acute oral toxicity in mice. Moreover, QACs, such as DDAB, are toxic to aquatic organisms but are safe for humans [34]. Many QACs are biodegradable under aerobic conditions [35]. Hence, GA in GD may play an important role in acute oral toxicity. Due to the low concentration of GD needed for application, we believe that this disinfectant can be considered safe and reliable.

### 3.5. Genetic Toxicity of GD* In Vitro*

In two test systems, i.e., with and without S9 metabolic activation, cytotoxicity indicators of RS, RSG, and RTG decreased as the dose of GD increased. The results indicated that GD is cytotoxic. In the S9 metabolic activation system (Table 3), the MFs of 4 doses of GD were approximately 1.16-fold, 1.32-fold, 0.87-fold, and 0.44-fold that of the solvent control, and the positive control was about 4.51-fold that of the solvent control. In the no activation system (Table 4), the MF of the positive control was about 4.54-fold that of the solvent control, and the MFs after application of 4 doses of GD were approximately 1.09-fold, 0.83-fold, 0.96-fold, and 0.47-fold that of the solvent control.

According to results evaluation by the China National Standard* in vitro mammalian cell TK gene mutation test* (GB 15193.20-2014), the RSG of the four GD concentrations were between 20% and 80%. The PE_0_ and PE_2_ of solvent control meet the standard requirements and there was a significant difference in MF between the positive sample and solvent control. The results indicate that the experiment was established in Chinese standard. However, the RTG of group 4 was less than 10% in the two systems, which did not meet the requirements of OECD 490. Therefore, it is considered that the GD mutation test needs to be improved. In addition, the MFs of using GD in the two systems did not exceed 3-fold that of the solvent control and did not increase when RS was under 20%. GEF was 103.5 and 64.8 in the presence and absence of S9 metabolic activation, respectively. Then it is required that the MF of either of the test concentrations should be higher than 229.6 and 190.8 according to GEF criterion for a positive result. However, the MF after GD treatment was less than 90 in our results. Thus, we recognized that the analysis of GD indicates a negative response, at least under our conditions of testing.

From Tables 3 and 4, it is found that the MF is low, the RS and RSG were about 20%, and RTG was less than 10% in group 4. Thus, we speculated that the GD damaged the cells. Actually, in the experimental process, we observed that the cells were still alive when they were added to microwells, but a large number of cells died over the culture period. The possible reasons for this phenomenon is beyond the scope of this study and requires further investigation.

### 3.6. Metal Corrosion of GD

Three metals (stainless steel, aluminum, and copper) were used to explore the metal corrosive properties of GD. The three treatments had no significant effect on the appearance of the three metals. As shown in Table 5, the corrosion rates of all three metals were less than 0.01, indicating that the three treatments did not corrode stainless steel, aluminum, or copper.

Metallic material is damaged by the action of the surrounding medium, which is known as metal corrosion. The mechanism of metal corrosion in different situations is complicated, and its main forms are chemical and electrochemical corrosion. The chemical reaction of metallic surfaces with the surrounding medium causes chemical corrosion. Metallic materials (alloys or impure metals) are contacted with electrolyte solutions to produce electrochemical corrosion through electrode reactions. The essence of metal corrosion is an oxidation process whereby electrons are lost.

GA, an aliphatic dialdehyde, has strong reducibility and relatively weak oxidation properties. DDAB, belongs to the family of cationic surfactants and probably functions as an inhibitor of corrosion in metal surfaces because cationic surfactants can be used as a kind of corrosion inhibitor. Cationic surfactants can be adsorbed onto the metal surface to form a protective film thereby changing the metal surface state and the electric double layer structure, thus enhancing the activation energy of the metal ionization process and producing a negative catalytic effect [36]. Hence, this is one of the main reasons for the low corrosiveness property of GD.

## 4. Conclusions

The results of bactericidal efficacy assays, toxicity assays, and metal corrosion tests showed that GD is effective and safe and causes low corrosion. It is important to provide theoretical support to develop a high-efficiency, low toxicity, and weakly corrosive disinfectant. In the future, we will study GD's ability to kill viruses. Moreover, fungi, viruses, and yeast will be investigated as experimental subjects to elucidate the bactericidal mechanism of general disinfectant.

## Figures and Tables

**Table 1:** Effective bactericidal concentration of five microorganisms.

Strains	Disinfectant	Treatment time (min)
*E. coli*	0.005%GA + 0.001%DDAB	10
*P. aeruginosa*	0.01%GA+0.002%DDAB	5
*S. aureus*	0.01%GA+0.002%DDAB	10
*C. albicans*	0.01%GA+0.002%DDAB	10
*A. niger *conidia	0.075%GA+0.015%DDAB	40

**Table 2:** Acute peroral toxicity assays of GD.

Groups	Dose	Number of mice	Number of dead mice	Growth rate of weight (%)	Mortality (%)
1	0	10	0	44 ± 1.3	0
2	0.05% GA + 0.01% DDAB	10	0	43.9 ± 3.0	0
3	0.1% GA + 0.02% DDAB	10	0	31.3 ± 2.8	0
4	0.2% GA + 0.04% DDAB	10	3	33.1 ± 2.3	30
5	0.4% GA + 0.08% DDAB	10	7	-1.56 ± 8.4^*∗∗*^	70
6	0.5% GA + 0.1% DDAB	10	10	- -	100

Note: *∗∗*  *P* < 0.01.

**Table 3:** Toxicity and mutagenicity of GD in mouse lymphoma cells in S9 metabolic activation.

Groups	PE_0_ (%)	RS_0_ (%)	PE_2_ (%)	RS_2_ (%)	RSG (%)	RTG (%)	MF (10^−6^)
solvent control	71.9	100.0	75.2	100.0	100.0	100.0	63.8
1	67.1	93.4	71.9	96.5	82.7	79.8	73.8
2	61.3	85.3	70.2	94.3	77.0	72.6	84.3
3	53.5	74.4	58.6	78.6	73.4	57.7	55.7
4	13.3	18.5	19.4	26.0	24.1	6.3	27.8
positive control	46.5	64.7	51.1	68.6	49.7	34.1	287.9^*∗∗*^

**Table 4:** Toxicity and mutagenicity of GD in mouse lymphoma cells in the absence of S9 metabolic activation.

Groups	PE_0_ (%)	RS_0_ (%)	PE_2_ (%)	RS_2_ (%)	RSG (%)	RTG (%)	MF (10^−6^)

solvent control	73.5	100.0	77.0	100.0	100	100	61.6
1	68.7	93.5	75.2	97.5	76.3	74.4	67.5
2	61.3	83.5	68.7	89.2	69.2	61.7	51.2
3	59.9	81.5	65.6	85.2	64.7	55.1	58.8
4	15.9	21.7	19.4	25.5	23.2	5.9	29.1
positive control	31.1	42.3	35.5	46.1	63.7	29.4	279.8^*∗∗*^

Note: solvent control is sterile water; positive control of S9 metabolic activation system is 3 *μ*g mL^−1^ cyclophosphamide; positive control of no activation system is 10 *μ*g mL^−1^ methyl methanesulfonate; Groups 1-4 are 50 *μ*g mL^−1^ GA + 10 *μ*g mL^−1^ DDAB, 100 *μ*g mL^−1^ GA + 20 *μ*g mL^−1^ DDAB, 150 *μ*g mL^−1^ GA + 30 *μ*g mL^−1^ DDAB, and 200 *μ*g mL^−1^ GA + 40 *μ*g mL^−1^ DDAB, respectively. *∗∗*  *P* < 0.01.

**Table 5:** Corrosion degree of sheet metal after GD treatment.

Sheet metal	water	0.005% GA + 0.001% DDAB	0.05% GA + 0.01% DDAB
	R (×10^−3^ mm a^−1^)	R (×10^−3^ mm a^−1^)	R (×10^−3^ mm a^−1^)
Stainless steel	5.3 ± 0. 08	0. 91 ± 0. 07*∗∗*	1.40 ± 0. 35*∗∗*
Aluminum	0. 57 ± 0. 60	0. 23 ± 0. 47	2.03 ± 1.48*∗∗*
Copper	3.69 ± 0. 13	7.25 ± 1.53*∗∗*	2.33 ± 1.41

Note: the means ± SD for at least three replicates are illustrated. *∗∗*  *P* < 0.01.

## Data Availability

The data used to support the findings of this study are available from the corresponding author upon request.
